# Application of a deep learning-based image analysis and live-cell imaging system for quantifying adipogenic differentiation kinetics of adipose-derived stem/stromal cells

**DOI:** 10.1080/21623945.2021.2000696

**Published:** 2021-11-19

**Authors:** Patrick Terrence Brooks, Lea Munthe-Fog, Klaus Rieneck, Frederik Banch Clausen, Olga Ballesteros Rivera, Eva Kannik Haastrup, Anne Fischer-Nielsen, Jesper Dyrendom Svalgaard

**Affiliations:** Department of Clinical Immunology, Rigshospitalet, Copenhagen University Hospital, Copenhagen, Denmark

**Keywords:** Adipogenesis, differentiation, adipose-derived stem cells, stem cells, deep learning, machine learning

## Abstract

Quantitative methods for assessing differentiative potency of adipose-derived stem/stromal cells may lead to improved clinical application of this multipotent stem cell, by advancing our understanding of specific processes such as adipogenic differentiation. Conventional cell staining methods are used to determine the formation of adipose areas during adipogenesis as a qualitative representation of adipogenic potency. Staining methods such as oil-red-O are quantifiable using absorbance measurements, but these assays are time and material consuming. Detection methods for cell characteristics using advanced image analysis by machine learning are emerging. Here, live-cell imaging was combined with a deep learning-based detection tool to quantify the presence of adipose areas and lipid droplet formation during adipogenic differentiation of adipose-derived stem/stromal cells. Different detection masks quantified adipose area and lipid droplet formation at different time points indicating kinetics of adipogenesis and showed differences between individual donors. Whereas *CEBPA* and *PPARG* expression seems to precede the increase in adipose area and lipid droplets, it might be able to predict expression of *ADIPOQ*. The applied method is a proof of concept, demonstrating that deep learning methods can be used to investigate adipogenic differentiation and kinetics *in vitro* using specific detection masks based on algorithm produced from annotation of image data.

## Introduction

Human adipose-derived stem/stromal cells (ASCs) are a mesodermally derived multipotent stem cell type with the ability to differentiate into mesenchymal derived cell types, i.e. adipocytes, chondrocytes and osteocytes, and can also differentiate into cells from other germinal layers[1,2]. ASCs are currently used in various clinical trials [clinicaltrials.gov] owing to their regenerative ability, immune and inflammatory process modulation, and other properties such as neovascularization and apoptosis reduction [3,4]. Understanding the properties of ASC adipogenic differentiation among individuals could help to improve clinical treatments, where fat grafts are applied e.g. for soft tissue reconstruction surgery after injury, chronic wounds or after cancer surgery/treatment. Preclinical and clinical trials have demonstrated that enriching fat grafts with ASCs or ASC-derived exosomes/extracellular vesicles can improve the survival and quality of the implanted fat grafts as a therapeutically active filler material [5]. In this setting, a likely mechanism of action of ASCs, apart from the paracrine effect, is that ASCs are more resistant to hypoxia than mature adipocytes and thus better survive the grafting procedure [6].

ASCs can be isolated from adipose tissue by enzymatic digestion and subsequent culture expansion of the released stromal vascular fraction (SVF), to enrich the ASCs [7]. Different culture conditions, especially the supplemented growth factors, can affect post-culture cell characteristics [8], including the ability of ASCs to differentiate into adipocytes [9,10]. Thus, there is a general need for proper potency assays that can accurately assess the differentiative capabilities of cultured cells. A better understanding of the differentiation kinetics of ASC adipogenesis through improved monitoring of lipid formation may eventually lead to better clinical products. Lipid droplet formation occurs during lipogenesis and fat generation, where they bud from the endoplasmic reticulum and contain a core of neutral lipids such as triacylglycerol (TAG) and cholesterol [11]. Lipid droplets can combine to form larger droplets, and adipocytes and hepatocytes can contain giant lipid droplets [12]. Lipid droplet formation and accumulation are processes that can be monitored using morphological data obtained through conventional microscopy [13]. The generation of lipids during adipogenesis is a complex process regulated by the gene expression of peroxisome proliferator-activated receptor γ (*PPARG), CEBPA/CEBPB* [14] and *ADIPOQ*, which leads to the production of adiponectin, an adipokine involved in adipocyte differentiation [15].

Adipogenic differentiation has traditionally been investigated by histological staining with fat soluble dyes such as Oil Red O (ORO), which stain lipids and triglycerides, but results are often merely reported as qualitative data. The standard method to quantify ASC adipogenic potency is photometric absorbance measurements of the ORO-stained lipid content in differentiated adipocytes. However, this method is prone to error owing to the many processing steps involved, which may lead to imprecise measurements and ORO has also been shown to stain non-adipocyte cells and preadipocytes [16]. To improve the detection of adipocytes and lipid content, recent studies have shown that ORO fluorescence detection can be dispensed with by directly analysing the area stained by dyes or antibodies that use different approaches for quantitative image analysis [17–19]. Other methods for quantifying adipogenesis in mesenchymal stem cells involve assays such as ELISA [20]. However, all current methods are time consuming and prone to human observational error [21].

To simplify cell trait quantification, the emergence of artificial intelligence-based methods that can recognize cell features has presented the opportunity to replace currently used methods. Different applications of deep learning (DL), a type of machine learning, seem promising for analysis of microscopy images [22]. Algorithms have been trained to detect cells of interest other than ASCs, either by human observation or guided by images of fluorescence-labelled cells [23,24]. DL algorithms have been used to accurately detect cellular properties during adipogenesis [22,25,26], and an approach to quantification of adipose tissue using deep learning based detection of ORO stained areas during differentiation has recently been described [27]. However, quantifiable analysis of the dynamics of differentiation has not yet been investigated using machine learning tools.

The methods needed to quantify and analyse processes by, for example, comparison with other process properties such as genetic information and developmental kinetics, are still needed to further validate the application of DL tools in cell biology and regenerative medicine. This study presents a method for the quantitative analysis of ASC adipogenic differentiation potency using a commercial DL tool (Cellari ApS) and live-cell imaging.

## Results

### Selection of adipose area detection mask

**Figure 1.**
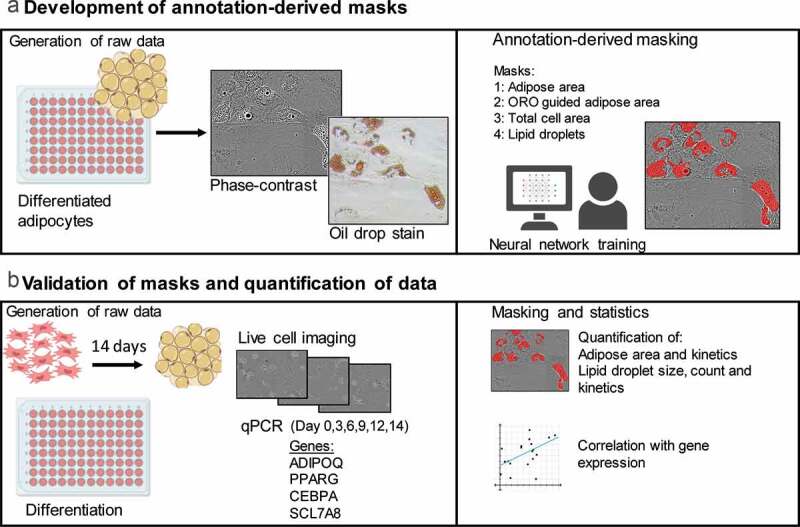
Experimental setup. (a) Adipose-derived stem cells (ASCs) underwent a 14-day adipogenic differentiation in a live-cell imaging system. The development of annotation masks was performed using images of cells and the generated masks were used to detect adipose area (Mask 1), ORO-guided adipose area (Mask 2), total cell area (Mask 3) and lipid droplets (Mask 4). (b) The generated detection masks were used to examine and quantify adipogenic differentiation in images obtained from live-cell imaging (day 0 – day 14). qPCR was used to analyse the expression of genes related to adipogenesis (*ADIPOQ, PPARG, CEBPA* and *SCL7A8*) at days 0, 3, 6, 9, 12 and 14 and possible correlation to adipose area and lipid droplet formation kinetics. The data represents three donors

**Figure 2.**
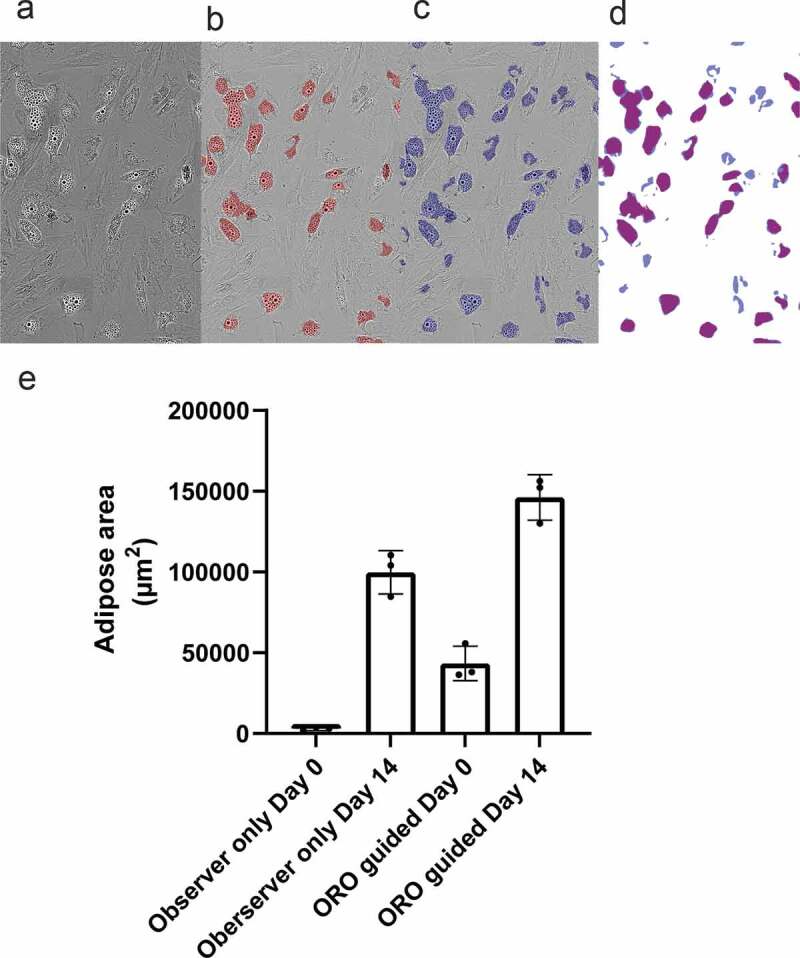
Adipose area mask results. (a) Phase-contrast image of adipocytes without masks. (b) Mask 1 – annotation of adipose areas by human observations alone. (c) Mask 2 annotation of by comparison to bright field images of ORO-stained areas found in the same 96-well after staining. (d) Overlay of Mask 1 and Mask 2, showing additional areas detected when using Mask 2. (e) Quantification of adipose area detected by Mask 1 and Mask 2 at day 0 and day 14 of differentiation. The data represents three donors. A significant difference between the means of Mask 1 and Mask 2 for both day 0 (p < 0.05) and day 14 (p < 0.05) was observed

After a 14-day adipogenic differentiation protocol using ASCs, annotation, training and generation of masks was completed (Figure 1) and adipose area was detected by Mask 1 (observer only, Figure 2a) or Mask 2 (ORO-guided annotations, Figure 2b) on images from Donor 1 on day 14. An overlay of Mask 1 and Mask 2 illustrates the differences in their estimation of adipose cells areas (Figure 2d).

The mean adipose area detected by Mask 1 at day 0 and day 14 was 2.8 × 10^3^ ± 1.6 x 10^3^ µm^2^ and 99.8 × 10^3^ ± 20.8 x 10^3^ µm^2^, respectively (Figure 2e). In comparison the mean adipose area detected by Mask 2 at day 0 and day 14 was 43.4 × 10^3^ ± 7.9 x 10^3^ µm^2^ and 146.2 × 10^3^ ± 27.0 × 10^3^ µm^2^, respectively (Figure 2e). The detected mean adipose cell area using Mask 2 was significantly larger for each of the donors compared to Mask 1 for both day 0 (p < 0.05) and day 14 (p < 0.05). The detection with Mask 2 may be more sensitive, but this mask may also detect nonadipocyte vesicles at day 0 and give an incorrect high background. Mask 1 was preferred over Mask 2 because of the lower detection at day 0.

### Quantification of adipose areas and lipid droplets

**Figure 3.**
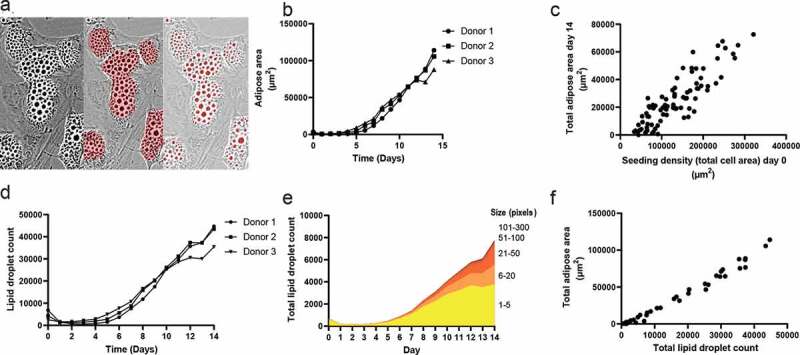
Adipogenic differentiation kinetics. (a) Example of adipose area (Mask 1) and lipid droplet (Mask 4) detection. (b) Detection of adipose cell area during the 14 days of differentiation. (c) Correlation between seeding density at day 3 and adipose cell area at day 14, r = 0.85, p < 0.001. (d) Lipid droplet formation during the 14 days of differentiation. (e) Distribution of lipid droplet size during differentiation. (f) Correlation between lipid droplet formation and adipose area, r = 0.98, p < 0.001

Adipose area and lipid droplet count and size was investigated to assess differentiation kinetics (representative detections by Mask 1 (middle) and Mask 4 (right) in Figure 3a).

An increase in adipose area was detectable on days 3–6 (Figure 3b). The mean adipose areas per well at day 14 for donor 1, 2 and 3 were 110.5 × 10^3^ µm^2^ ± 23.0 x 10^3^ µm^2^, 104.2 × 10^3^ µm^2^ ± 11.1 x 10^3^ µm^2^ and 84.8 × 10^3^ µm^2^ ± 28.1 x 10^3^ µm^2^ respectively. The mean areas increased until day 14, except for Donor 3, where a plateau around day 12 was seen before increasing again until day 14.

A comparison of adipose area at day 14 (Mask 1) to the total cell area at day 0 (Mask 3) for each image taken from the wells for all three donors was performed to assess the relationship between seeding density and the resulting detected adipose area after differentiation (Figure 3c). There was a significant correlation between the cell-covered area at day 0 and the amount of detected adipose area at day 14 (p < 0.0001).

The number of lipid droplets increased at approximately day 3–5 (Figure 3d). There was a temporary plateau at approximately days 11–12 for 2–3 days after which the number of lipid droplets increased again until day 14. Lipid droplet size shows accumulation of larger size vesicles as differentiation progresses (Figure 3e). Results indicate considerable interdonor variation in the number and size (Supplemental Figure 2). Additionally, the lipid droplet count was significantly positively correlated with the adipose area size (p < 0.0001, Figure 3f).

### Adipogenic gene expression and comparison to deep learning detection data

**Figure 4.**
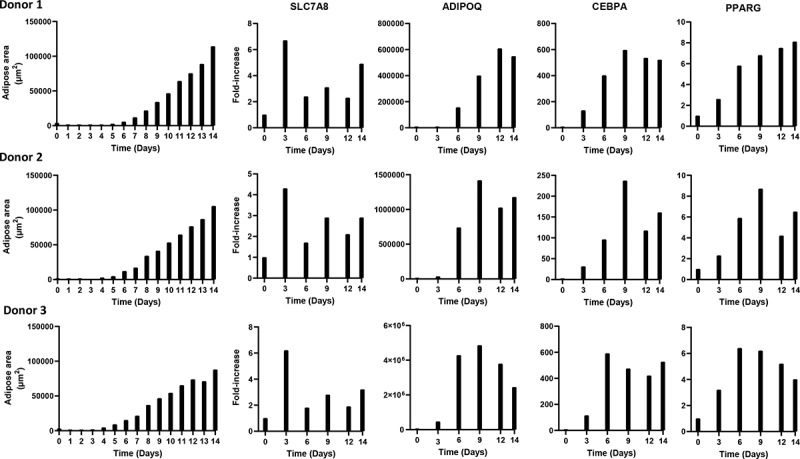
Adipose area per donor and adipogenic gene expression during the 14-day adipogenic differentiation

Quantitative PCR was used to evaluate the expression of several genes shown to be involved in adipogenic differentiation (Figure 4). *SLC7A8* expression was increased by up to 7-fold showing a similar expression pattern among the donors. The highest expression level for all donors was observed on day 3, and *SLC7A8* continued to be expressed at relatively low levels throughout differentiation. *ADIPOQ* was expressed from day 3, with maximum expression levels of 0.6–4.9 x 10^6^-fold. *CEBPA* expression increased around day 3, reaching an approximately 600-fold induction for Donor 1 and 3 and an approximately 250-fold for Donor 2 at days 6–9. *PPARG* expression was present from approximately day 3, with a maximum expression increase of 9-fold.

The qPCR data and the adipose area detected by the DL tool revealed increasing trends of adipose area and *ADIPOQ, CEBPA* and *PPARG* expression. However, there was no significant correlation between gene expression and the adipose area and lipid droplet detection results.

### Quantification of adipocyte differentiation by measuring oil red o absorbance

The ORO absorbance was quantified for all donors using the 96-well and 12-well plates for image acquisition (Supplemental Figure 1). No significant difference was observed when quantitating the fraction of adipogenically differentiated and nondifferentiated cells in the 96-well plate. The cell seeding density at 1:4 did not show a clear difference, but a clear difference was seen in the 12-well plate (Supplemental Figure 1).

## Discussion

In this study, we combined a live-cell imaging system with a DL tool to investigate and quantify the adipose area and lipid droplet formation during adipogenic differentiation of ASCs as a means of measuring differentiative potency. These data were compared to adipogenic gene expression to investigate a possible correlation between gene expression and DL-detected adipose area and droplet formation. The DL tool was able to successfully detect adipose areas either through annotation by human observation alone or guided by images from conventional ORO staining of differentiated adipocytes. The number of donors included is relatively small, and while the study has shown that the method is applicable, this limits the study’s predictive assessment of adipogenic differentiation dynamics and studies including more donors are warranted.

Quantifying the increase in adipose area enabled us to investigate differentiative kinetics of ASC adipogenic differentiation. There was a lag phase before the adipose area was detectable, which could correspond to the initial proliferative lag phase described for adipogenic differentiation as ASCs proliferate [28,29]. Interestingly, the differentiation kinetics throughout the differentiation period were close to similar for all three donors, until day 12–13 where a temporary plateau phase is observed. A closer look at the data showed that the number of detected objects (adipose areas) and their mean size decreased between day 12–13 for Donor 3 leading to a 30% drop in mean adipose area for 1 of 7 wells. This seems to arise from a combination of cell migration, that can lead to cells wandering from the image frame, as well as an observed visible minor frameshift, that happened in the IncuCyte system between day 12–13. If many cells with adipose content are located along the periphery it may have a measurable effect on the data outcome.

A continuous increase in the adipose cell area and lipid droplet count (Figure 3b, d) indicated that differentiation was not a synchronous event for all cells, which was supported by video imaging of the 14-day differentiation (Supplemental Figure 4). This could indicate that the nonsynchronous event of adipogenesis in ASCs could be further stratified by assessing the presence of ASC subtypes at different time points when using the heterogenic primary ASC population from the SVF. Whether the observed differences among donors regarding the adipose area and lipid droplets are caused by the presence of preadipocytes in the seeded ASC population or by intercell differences in the differentiation kinetics needs to be determined. This could be investigated by e.g. flow cytometry analysis for the presence of pre-adipocytes markers in the seeded cell population. The presence of granular/vesicular ASCs detected by the ORO-guided mask (Mask 2) at day 0 might indicate committed preadipocytes (Figure 2e). Future studies should investigate whether this is the case by combining DL with cell tracking software and single-cell characterization and quantification.

Variation in the ASC potential for adipogenic differentiation has previously been assessed by measuring the expression of genes involved in adipogenesis [30]. Donor variation in adipogenic potency is multifactorial, involving multiple effectors of adipogenic gene expression and phenotypical factors such as autocrine and paracrine molecules [31]. However, we only observed a minor difference in the day 14 adipogenic potential, quantified as the amount of detectable adipose area and lipid droplet content between the examined donor cells.

The data from day 14 of differentiation illustrate significant inter- and intra-well variation in the detected adipose area, which is emphasized by the positive correlation between the seeding density and the total cell area at day 0 (Mask 3) and the detected adipose area at day 14 (Mask 1). This demonstrates the importance of uniform seeding in the wells if the precision of this method is to be fully harnessed to detect small differences between donors or between individual wells. The use of conditioned media and the concentration of local paracrine factors may also play a role, as previously shown [32]. The inclusion of more donors might lead to demonstrable variation in adipogenic potency between individuals when using the applied experimental setup.

Regarding adipogenic gene expression, *ADIPOQ* has previously been shown to be induced between day 1 and day 7 [33]. We observed a similar result, with increased expression beginning between day 3 and day 6. We also found a similar expression profile for *CEBPA* and *PPARG*, both showing increased expression with an earlier onset than that of *ADIPOQ*. We observed a higher induction of *ADIPOQ* and *CEBPA* compared to what has been reported [33], which could reflect the fact that later passage cells (passage 6–15) were used in the study by Ambele et al.

The expression of *ADIPOQ, CEBPA* and *PPARG* increased at approximately the same time at which DL was able to detect the formation of adipose areas and lipid droplets. The lipid droplet detection tended to be more sensitive, as single droplets could be detected, whereas clusters of droplets were used to train the adipose area recognition.

Whereas *ADIPOQ, CEBPA* and *PPARG* are induced during adipogenesis, *SLC7A8* is expressed early in adipogenesis and has been found to decrease as ASCs start to differentiate [33]. We observed an initial induction of *SLC7A8* between day 0 and day 3, followed by a subsequent decline, which is different from that previously reported [33].

To verify that the masks were indeed able to recognize areas of interest, Mask 2 was trained with bright field microscopy and live cell images of ORO-stained differentiated adipocytes. When comparing Mask 1 and Mask 2 we found a difference between the detected adipose areas at day 14. As ORO staining simplifies the discrimination of cells with or without lipids, it should lead to a more sensitive mask. Although a similar increase in adipose detection from day 0 to day 14 was found for the two masks, a substantially higher fraction of adipose area was quantified with Mask 2 (ORO-guided) at day 0. Even with the ORO control to assist training, it may still be difficult to differentiate between small lipid droplets, background noise, and other cell structures at day 0. Mask sensitivity may therefore vary according to the person annotating and training the software and the staining methods applied as a control for areas of interest. A more consistent result might be achieved with more conservative training, yielding results based on clearly defined lipid droplets formed during differentiation. Detection masks might be improved by combining ORO staining and immunohistochemistry for automated verification of adipose droplets or other cellular components.

The use of conventional quantitative methods to assess adipogenic potency is often demanding and tiresome work if image data has to be annotated by an observer, where data acquired by observation using staining methods are prone to observer variation and bias [34]. The use of machine learning to quantify the adipose cell area has the potential to be much more sensitive than traditional absorbance measurements of ORO-stained cells and the loss of information in the acquired images due to sample processing (Supplemental Figure 3) can be bypassed. Our results show that 96-well plates, and potentially any setup that allows images to be obtained, are suitable for DL analysis, whereas absorbance quantification of conventional ORO-stained cells is not feasible in 96-well plates.

Microscopy images can now be compared and correlated with morphological changes and genetic expression profiles during differentiative processes. This could lead to new insights into the kinetics of these processes without the need for staining to verify cellular changes and to help generate new methods for improving cellular treatments in which ASCs are applied.

## Conclusion

We demonstrated that this approach was able to investigate and quantify the adipose area and lipid droplet kinetics during adipogenic differentiation. We also observed that the seeding density is important for improving the sensitivity. The large amount of cellular data on adipogenic properties that can be acquired by combining live-cell image acquisition and deep learning-based recognition could potentially lead to better methods for image-based quantification and eventually better cellular therapies.

Future studies are needed to validate this approach to quantify adipogenic potency. The method presented here forms the basis of a relatively easy to use setup for the mass evaluation of donor cell variation, for example in drug-screening experiments, and is a proof of concept for the use of deep learning methods to monitor adipogenic differentiation kinetics.

## Materials and methods

### Experimental setup

ASCs underwent a 14-day differentiation protocol in order to quantify and assess adipogenic differentiation and kinetics (Figure 1). Images obtained during adipogenesis were used to annotate regions or objects of interest in the DL tool, and algorithms were trained to detect the adipose area in cells (Mask 1 and Mask 2), total cell area (Mask 3) and lipid droplets (Mask 4) in live-cell images. These were compared with adipogenic gene expression of ASCs at different time points during differentiation to look for possible correlation to differentiation kinetics.

### SVF isolation and cell culture

Lipoaspirate was obtained from 3 healthy donors during cosmetic surgery. All donors were female Caucasians and were between the ages of 18 to 60 years. The surgical procedure was primarily focused on abdominal fat tissue where the Coleman technique was applied. The aspirated adipose tissue was removed using Klein’s solution (1 L NaCl; 400 mg xylocaine; 1 mg adrenalin) in volumes equivalent to the aspirated volume of adipose tissue, and at least 50 mL was aspirated in order to produce a viable stromal vascular fraction.

The stromal vascular fraction was harvested as previously described [35]. Briefly, lipoaspirate tissue was digested with collagenase (Nordmark, N0002880) at 37°C for 45–60 min under constant stirring. The collagenase was neutralized by adding cell culture media consisting of DMEM (Gibco™, 22,320,030) supplemented with 10% pHPL (produced at the Department of Clinical Immunology, Rigshospitalet [36]), 1% penicillin/streptomycin (Gibco**™**, 15,140–122), and 2 IE/mL heparin (Amgros I/S, Rigshospitalet, 741,827). The digest was filtered to remove undigested components and centrifuged for 10 min at 1200 x g. The cell pellet was resuspended in cell culture media, and the cells were counted using a NucleoCounter® NC-3000**™** (Chemometec) and Via-1 cassettes (Chemometec, 941–0012). SVF was seeded in cell culture media and expanded for 1–2 passages before use in the differentiation assay. Cell culture medium was changed every 1–2 days.

### Adipogenic differentiation

ASCs (passages 1–2) were seeded in 24-well or 96-well plates at two concentrations of 1 × 10^4^ and 2.5 × 10^3^ cells/cm^2^. Twenty-four hours after seeding, cell culture media was exchanged for differentiation media (StemPro**™** Adipogenesis Differentiation Kit (Gibco**™**, A1007001)). The adipogenic differentiation medium replaced every 3 days throughout the 14-day differentiation period. Images were captured at days 0 and 14 with the IncuCyte® (Sartorius) at 20x magnification. 8 wells were seeded per donor including a negative control well were the cells received culture media without differentiation supplements.

### Oil red O staining and absorbance measurements

Lipid droplets in differentiating adipocytes were stained with Oil Red O (Sigma Aldrich, O0625).

Briefly, the differentiation medium was removed, and the cells were washed once with PBS (Gibco**™**, 14,190–0949) before fixing in 4% paraformaldehyde for 30 min. The cells were then incubated in 60% isopropanol for 5 min. The cells were subsequently washed with distilled water and incubated for 5 min with an Oil Red O staining solution (1:3 dilution of 0.3% Oil Red O in isopropanol and distilled water), followed by three washes with distilled water. All steps performed at room temperature. Images of Oil Red O-stained cells were taken with an inverted microscope (Carl Zeiss).

Oil Red O was extracted from the cells by removing the distilled water and adding 99% isopropanol. Following thorough mixing, the isopropanol containing the extracted Oil Red O was transferred to a 96-well plate. The absorbance was measured at 492 nm, subtracting the absorbance from the pure isopropanol used for extraction.

### Gene expression by qPCR

Four genes related to adipogenesis were selected to elucidate a possible relationship with the adipose areas detected by the DL tool. A gene expressed in early adipogenesis (*SLC7A8*) was assessed using qPCR, along with three genes (*ADIPOQ, CEBPA*, and *PPARG)* that are expressed during the mid-to-late differentiation of fat cells.

Primers and probes were designed using Primer3web version 4.1.0. (Supplementary Table 1). The primers and probes were obtained from Eurofins Genomics. All primers were designed with a T_m_ of 60°C (± 1°C); probes were designed with a T_m_ of 69°C (± 1°C). The probes were labelled with FAM and TAMRA.

The gene targets were *B2M, PPARG, CEBPA, ADIPOQ* and *SLC7A8. B2M* was used as a calibrator for differential gene expression. The *CEBPA* gene did not contain any introns; for the remaining targets, the primers were designed to span an intron.

Total RNA was isolated from cells at days 0, 3, 6, 9, 12 and 14 during the differentiation period (RNeasy Kit, Qiagen). The RNA concentration was calculated assuming OD = 1 as equivalent to 40 µg/mL RNA at 280 nm. The OD ratio of 180/260 of the RNA measurements was close to 2, indicating high-purity RNA. Total RNA was used as a substrate for synthesizing cDNA using a First Strand cDNA Synthesis Kit (Thermo Scientific, K1622), random hexamer priming, and MMuLV reverse transcriptase in a total volume of 20 µL following the manufacturer’s recommendations.

Real-time PCR was performed using an ABI 7500 detection system (Applied BioSystems, Foster City, CA, USA) with TaqMan chemistry. For each sample, 2 µL of transcribed total RNA was used for PCR amplification in a total volume of 25 µL with 2x Universal Master Mix (Thermo Fisher), with a final primer concentration of 600 nM and a final probe concentration of 250 nM. The thermoprofile was 50°C for 2 min, 94°C for 10 min, 94°C for 30 sec, 60°C for 30 sec, and 72°C for 30 sec for a total of 40 cycles. The data were analysed using the 2^−Δ∆CT^ method with the *B2M* as a reference [37].

### Machine learning method, mask selection and adipose/lipid droplet detection

A commercial deep learning-based tool for image recognition (Cellari ApS) was applied to generate area data from the images acquired on days 0 and 14 of adipogenic differentiation by distinguishing areas with a lipid/adipose morphology and quantifying them. This software uses a convoluted neural network-based model [38] to detect areas of interest that are annotated by the user, creating detection algorithms or ‘masks’ that can be applied for image analysis.

Four different masks were generated by fat area and lipid droplet annotation on images taken from Donor 1 on Day 14. Approximately 6–8 hours were spent on annotating each mask. Mask 1 was annotated by human observations of cell areas considered to be adipose/lipid. For Mask 2 annotation was done by direct comparison of adipose areas from Day 14 images generated by the IncuCyte live-cell imaging to bright field images of ORO-stained areas found in the corresponding well after staining (Figures 2b, c). Mask 3 was trained to detect the total cell-covered area (not shown) by annotating all areas covered by nucleated cells excluding cellular debris. Finally, Mask 4 was trained to detect lipid droplets by annotating intracellular lipid droplets situated in adipose areas (Figure 3a).

The mask sensitivities and their ability to detect the adipose area were compared to choose the optimal mask for use in the quantification of adipose area kinetics (further described in the Results section). Mask 1 and Mask 4 were applied to serial images from days 0 to 14 acquired from each donor to quantify the kinetics of the adipose area and lipid droplets during adipogenic differentiation. Adipose area means were converted from pixels to µm^2^ using manufacturer specifications (at 20x magnification 0.62 µm/pixel; 1 pixel = 0,3844 µm^2^).

### Statistics

Correlation between increase in adipose area and lipid droplet count, and Mask 1 and 3, was analysed using Spearman’s correlation. Means and standard deviations were calculated to plot adipogenic kinetics and a comparison Mask 1 and Mask 2 means on day 0 and 14 was calculated using a paired parametric t-test using GraphPad Prism 9 (GraphPad Software, Inc.).

## Supplementary Material

Supplemental Material

## Data Availability

Raw data was generated at the Department of Clinical Immunology, Rigshospitalet, Copenhagen University Hospital, Denmark. Derived data supporting the findings of this study are available from the corresponding author P.T.B. upon request.
