# Supplementary material for: Application of a deep learning-based image analysis and live-cell imaging system for quantifying adipogenic differentiation kinetics of adipose-derived stem/stromal cells
Source: Adipocyte. 2021 Nov 19;10(1):621–30. doi: 10.1080/21623945.2021.2000696 (PMC8632106; doi:10.1080/21623945.2021.2000696)
Supplement: Supplemental Material [file KADI_A_2000696_SM8319.zip › supplementary/Supplemental legends.docx]

**Supplemental Table 1:** qPCR primers

**Supplemental Figure 1:** Conventional Oil Red O fluorescence quantification of adipogenesis

**Supplemental Figure 2:** Lipid droplet kinetics

**Supplemental Figure 3:** Loss of cell information during the staining process

**Supplemental Figure 4:** Differentiation video – Adipogenic differentiation (Donor 1, day 0 to 12)
