# Supplementary figures and images for: Application of a deep learning-based image analysis and live-cell imaging system for quantifying adipogenic differentiation kinetics of adipose-derived stem/stromal cells
Source: Adipocyte. 2021 Nov 19;10(1):621–30. doi: 10.1080/21623945.2021.2000696 (PMC8632106; doi:10.1080/21623945.2021.2000696)

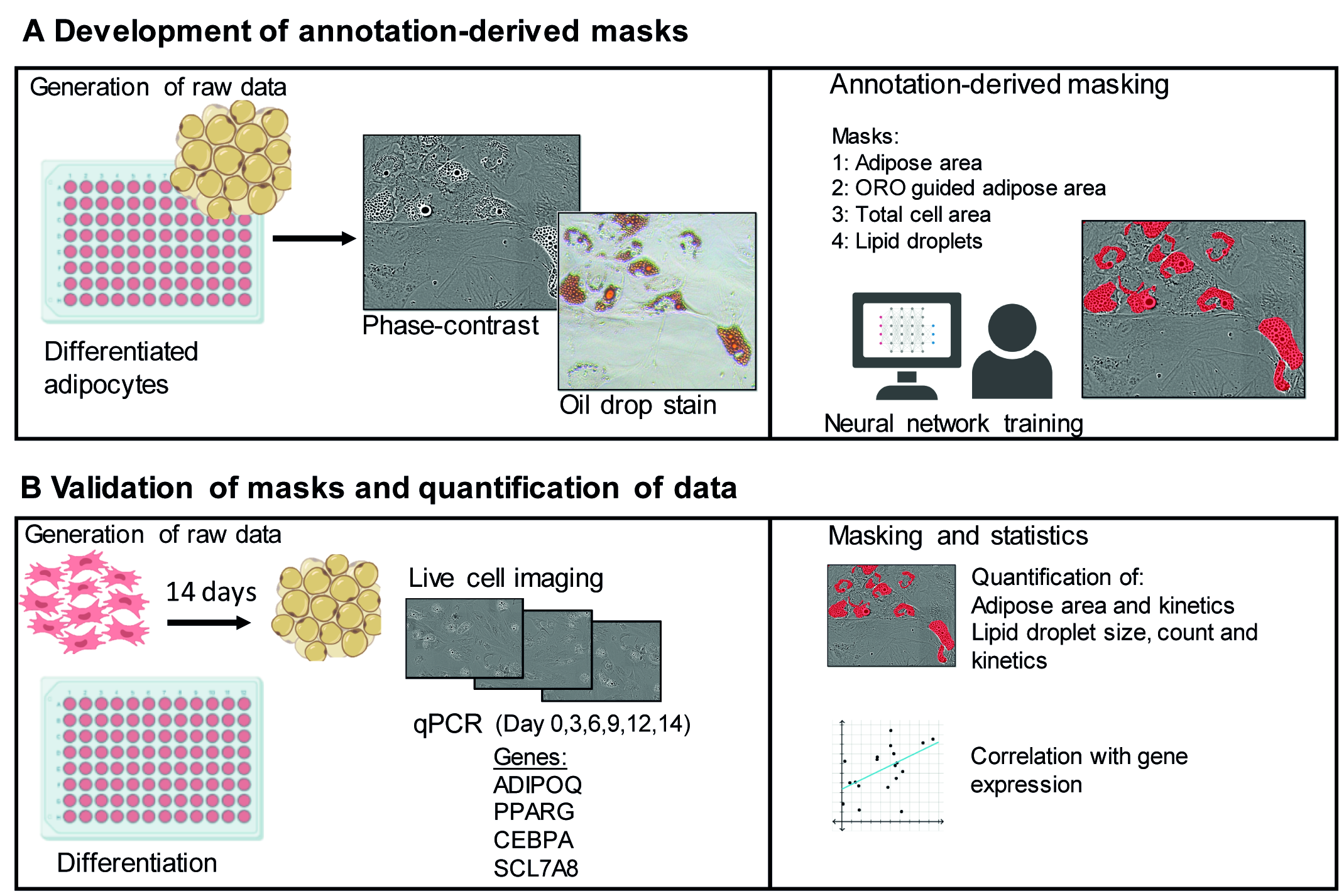

Supplement: Supplemental Material [file KADI_A_2000696_SM8319.zip › supplementary/Supplemental_Figure_1.tif]

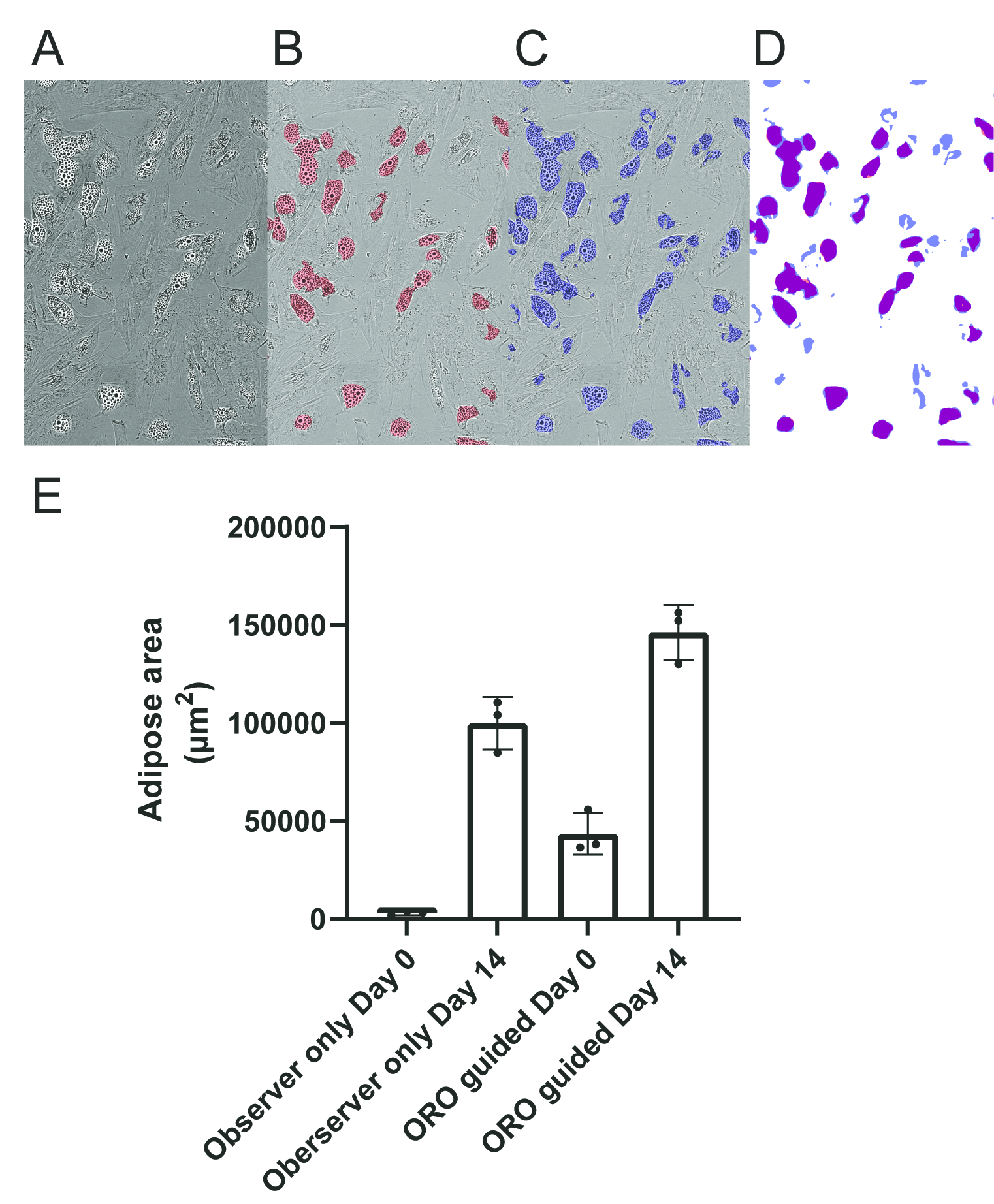

Supplement: Supplemental Material [file KADI_A_2000696_SM8319.zip › supplementary/Supplemental_Figure_2.tif]

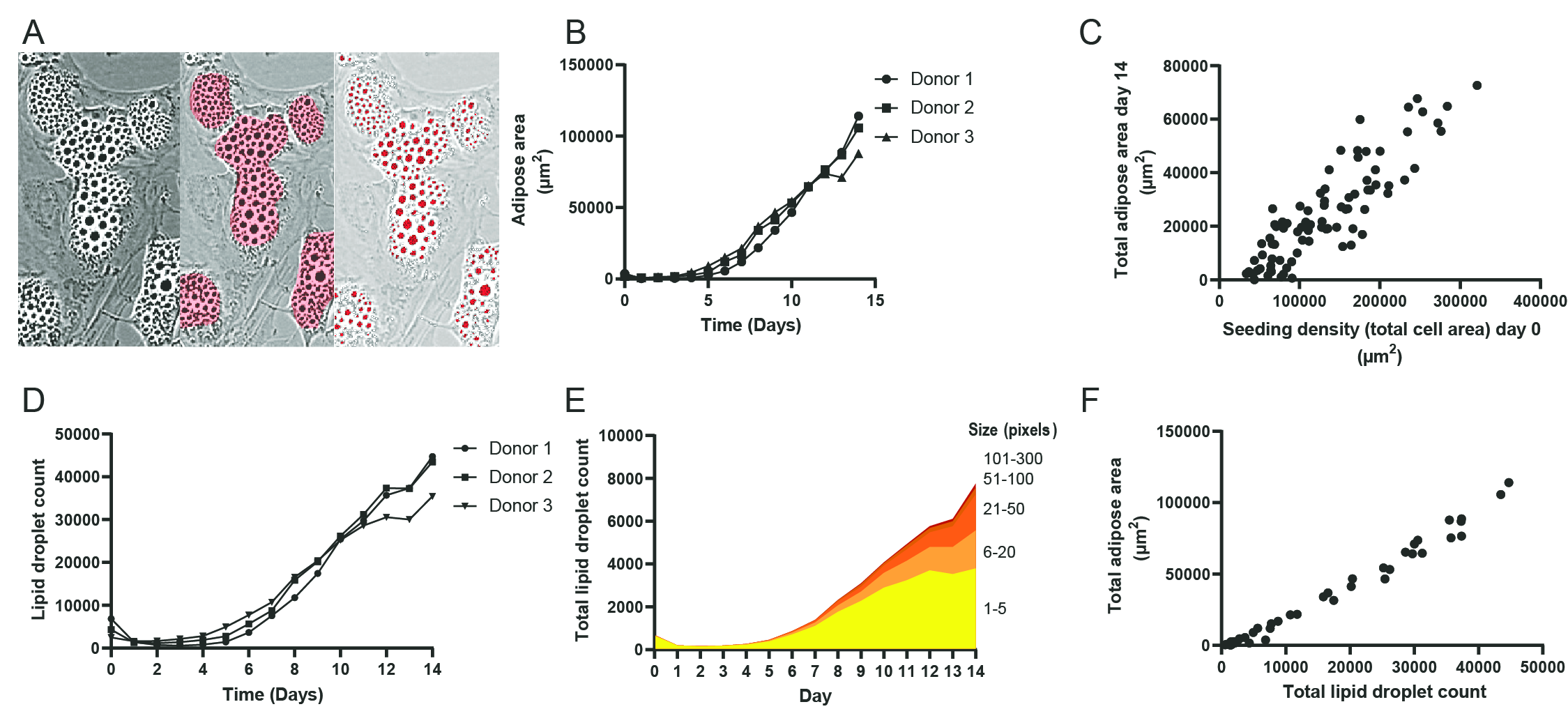

Supplement: Supplemental Material [file KADI_A_2000696_SM8319.zip › supplementary/Supplemental_Figure_3.tif]
